# Navigating the network: a narrative overview of AMR surveillance and data flow in the United States

**DOI:** 10.1017/ash.2024.64

**Published:** 2024-04-19

**Authors:** Darin W. Robillard, Alexander J. Sundermann, Brian R. Raux, Andrea M. Prinzi

**Affiliations:** 1 Division of Public Health, University of Utah School of Medicine, Salt Lake City, UT, USA; 2 Corporate Program Management, bioMérieux, Salt Lake City, UT, USA; 3 Division of Infectious Diseases, University of Pittsburgh School of Medicine, Pittsburgh, PA, USA; 4 US Medical Affairs, bioMérieux, Salt Lake City, UT, USA

## Abstract

The antimicrobial resistance (AMR) surveillance landscape in the United States consists of a data flow that starts in the clinical setting and is maintained by a network of national and state public health laboratories. These organizations are well established, with robust methodologies to test and confirm antimicrobial susceptibility. Still, the bridge that guides the flow of data is often one directional and caught in a constant state of rush hour that can only be refined with improvements to infrastructure and automation in the data flow. Moreover, there is an absence of information in the literature explaining the processes clinical laboratories use to coalesce and share susceptibility test data for AMR surveillance, further complicated by variability in testing procedures. This knowledge gap limits our understanding of what is needed to improve and streamline data sharing from clinical to public health laboratories. Successful models of AMR surveillance display attributes like 2-way communication between clinical and public health laboratories, centralized databases, standardized data, and the use of electronic health records or data systems, highlighting areas of opportunity and improvement. This article explores the roles and processes of the organizations involved in AMR surveillance in the United States and identifies current knowledge gaps and opportunities to improve communication between them through standardization, communication, and modernization of data flow.

## Introduction

Antimicrobial resistance (AMR) has significant global health implications, with an estimated 4.95 million deaths in 2019 alone.^1^ AMR surveillance, crucial for understanding and preventing the spread of AMR, involves collecting, analyzing, and reporting data on organism susceptibility patterns. Effective strategies against AMR, including enhanced infection control, vaccination, and judicious antimicrobial use, benefit from comprehensive surveillance data.^1^ In the United States, diverse surveillance programs led by national or state public health organizations involve clinical, public health, and national reference laboratories.^2^ Although many of these programs are established, how data are collected, shared, analyzed, and reported for each program is poorly understood. For this review, we reviewed publicly available databases, AMR surveillance literature from the United States, and performed informational interviews^3–7^ with various organizations to better describe the current surveillance landscape. We aim to provide a comprehensive overview of the state of AMR surveillance in the United States and identify potential barriers and opportunities for improvement in data sharing, completeness, standardization, and overall surveillance efforts.

## The role of clinical laboratories in susceptibility testing and AMR surveillance

The clinical microbiology laboratory is responsible for performing antimicrobial susceptibility testing (AST), which generates the data necessary to appropriately treat infections. Although the primary purpose of the clinical microbiology laboratory is providing data to inform patient care, it is also a rich source of AST data. These data can be utilized not only for monitoring AMR trends but also for informing public health initiatives.^8,9^ The level of participation a clinical laboratory has in active or passive AMR surveillance will vary depending on local policies and procedures, laboratory capacity, the need for data to answer a particular public health question, and the level of collaboration with public health and national laboratories or other national surveillance programs.^9^ However, even in high-income countries, coalescing and sharing microbiology data is heavily dependent on infrastructure and resources.^10^ To date, comprehensive information on how clinical laboratories aggregate their AST data and participate in public health surveillance is absent, highlighting a knowledge gap in fully understanding the AMR surveillance landscape.

A primary challenge in understanding AST data collection and sharing from the clinical laboratory is variability in practice. Various methodologies are used to perform AST including manual methods (eg, disk diffusion, gradient diffusion, agar dilution, and broth microdilution), automated methods, and genotypic detection of resistance mechanisms.^8,11^ In addition to the variation and error that may occur between methods,^12^ there is significant variability in how laboratories process and perform bacterial culture, as well as how the workup (including AST) is performed.^13^ These challenges are further exacerbated by variability in the use of AST interpretative criteria, also known as breakpoints.^14^

Susceptibility testing yields minimum inhibitory concentrations (MICs) (obtained via broth microdilution or gradient diffusion) or zones of inhibition (from disk diffusion testing). Breakpoints, based on MIC distribution and clinical data, categorize AST data into categories that correlate with clinical outcomes.^15^ This categorization is vital for patient care, AMR surveillance, and policy development. The United States uses 3 primary standard development organizations (SDOs): Clinical and Laboratory Standards Institute (CLSI), Food and Drug Administration (FDA), and the European Committee for Antimicrobial Susceptibility Testing (EUCAST). Notably, although it is not currently an SDO, the US Committee on Antimicrobial Susceptibility Testing (USCAST) is a EUCAST-affiliated committee that also establishes testing and interpretation standards. In the United States, clinical laboratories can choose to use breakpoints from the SDO of their choice, and the reasons for different breakpoint applications are complex.^16^ Furthermore, the use of outdated criteria may affect the interpretation of AMR epidemiology.^14^ Recent regulations by the College of American Pathologists (CAP), a major laboratory accrediting body in the United States, mandate the use of updated breakpoints for all AST methods within 3 years of the breakpoint publication date. Although this action is crucial for ensuring that AST interpretation is accurate, performing these updates is a complex process that regulatory hurdles, manufacturing capabilities, and limited laboratory staffing may affect. Given these challenges, variability in the use of updated breakpoints persists.

Susceptibility test data may be collected, stored, and summarized in a laboratory information system (LIS), electronic health record system (EHR), data analytics software, or a combination of these. The lack of standardized formats and interoperability poses challenges.^17^ Some AMR surveillance programs have specific data submission requirements, creating potential barriers.^18^ The process for aligning with data requirements can be complex and may involve additional validation steps for the laboratory, which may limit participation given existing staffing and capacity challenges. The World Health Organization (WHO)’s WHONET, available in 44 languages, serves as a model for overcoming these barriers by offering free software with modules for laboratory functions, data analysis, reporting, and data encryption. The WHONET software also standardizes data from laboratory instruments and LIS, facilitating global AMR surveillance in 130 countries.^19^

The era of advanced diagnostic microbiology testing has ushered in novel mechanisms for collecting and sharing AST data. For example, some medical device companies have enabled cloud database functionality with their devices, such as multiplex polymerase chain reaction testing platforms that test for various infections.^20^ These data can be accessed from the cloud if laboratories are willing to share their de-identified test data. This capability allows for the harnessing of near real-time surveillance data which can aid in tracking spatiotemporal trends in resistance. This introduction of advanced diagnostics and informatics has also expanded antibiogram capabilities. Although routine cumulative antibiograms are used to guide clinicians in the selection of empiric therapy, multi-facility antibiograms can be used to understand AMR at a health system or regional level.^21^ In many settings, the creation of a routine cumulative antibiogram is mandated, and this may provide ample site-specific data that can be aggregated for public health use. It is important to note that antibiograms may be compiled using data derived from various sources (eg, AST instrument, EHR, and LIS). Due to this, the data quality should be scrutinized prior to use in the antibiogram. Finally, the roles of clinical and public health laboratories differ significantly. Effective communication both within and among these laboratories is crucial for efficient data sharing. Clinical laboratories participating in this effort must establish algorithms to alert staff when unusual resistance patterns are detected, along with clear guidelines on the subsequent steps to be taken. This may involve in-house confirmatory resistance mechanism testing, subculturing and sending isolates to the local public health laboratory for additional testing, sending raw AST data to the public health department to support AMR surveillance efforts, or a combination of these actions.^22^ Understanding these complexities is essential in standardizing clinical laboratory data for representative analysis and surveillance.

## AMR surveillance at local and regional public health levels

In the United States, the Centers for Disease Control and Prevention (CDC) Antibiotic Resistance Laboratory Network (ARLN) facilitates data and testing transfer between clinical and public health laboratories nationwide. The ARLN, spanning labs in all 50 states, bridges the gap in local laboratory capabilities for effective AMR surveillance and prevention.^22^ Clinical laboratories develop protocols to identify unusual resistance in routinely tested organisms, coordinating with public health labs on subsequent isolate and specimen testing. At the public health laboratory level, test methods include phenotypic AST, genotypic resistance gene detection, or phenotypic confirmation of specific resistance mechanisms. Results from confirmatory testing are communicated back to the clinical laboratory for patient management. Isolates with unusual resistance patterns undergo additional testing at regional public health laboratories, which also support CDC-funded AMR surveillance programs. Regional labs conduct testing, including whole genome sequencing (WGS), for initiatives like the Gonococcal Isolate Surveillance Project (GISP) and the National Tuberculosis center program. They are also responsible for multidrug-resistant organism screening from healthcare facility specimens.

## Examples in action: Wisconsin Clinical Laboratory Network (WCLN) and the California Reportable Disease Information Exchange (CalREDIE)

Exemplary collaborations in the United States for AMR surveillance include the WCLN formed in 2001, involving 138 laboratories.^23^ The success of the program is strongly rooted in 2-way communication, training, and education. In 2014, the network aimed to create a statewide antibiogram by compiling data from 72 WCLN facilities. Ultimately, the analysis involved more than 150,000 organism–antimicrobial combinations.^24^ Through this process, WCLN identified limitations in local testing practices and data standardization, leading to the creation of the Surveillance of Wisconsin Organisms for Trends in Antimicrobial Resistance and Epidemiology (SWOTARE) program. Through this structure, laboratories in each WCLN region send clinically important organisms to a centralized testing facility which uses reference broth microdilution AST to compare resistance profiles by location and time.^23,25^

CalREDIE, implemented by the California Department of Public Health (CDPH), started as a pilot in 2010.^26^ During the successful pilot period, CDPH and participating jurisdictions had access to real-time laboratory reporting. Since the completion of the pilot, all local public health departments in California have begun participating in the program in some capacity, and CDPH continues the iterative process of adding modules and making improvements to the system based on surveillance needs.^26^ CalREDIE’s success lies in a centralized database and modules supporting data sharing from providers and clinical laboratories. The electronic laboratory reporting module allows secure transmission of standardized results, replacing manual entry and supporting Health Level 7 compliance.^27^ A 2023 publication demonstrates the program’s effectiveness in characterizing Gonococcal infection trends in California between 2020 and 2021, influencing programmatic interventions and clinical recommendations.^28^

## AMR surveillance at the national level

### CDC NARMS

The National Antimicrobial Resistance Monitoring System (NARMS) is an interagency surveillance system maintained by the CDC, FDA, and US Department of Agriculture (USDA). Its focus lies on foodborne and other enteric bacteria in humans, retail meats, and food animals. NARMS receives clinical specimens (and isolates, depending on the pathogen) from public health laboratories. These data are available to the public via reports that provide visual access to AST results and trends (Table 1).

**Table 1.** AMR surveillance program characteristics, United States

Program	Organization	Selection of participating labs	How participation is achieved	Data submission frequency	Type of data submitted	Breakpoints standard used	Data handling	Data sharing	Challenges/barriers
ATLAS (global in scope with US participation)	Pfizer	Typically, long-standing relationships. Field representatives (Pfizer) may also recommend sites; IHMA helps vet potential site	Voluntary, through partnership	Several times a year, in batches. Approximately 280 isolates submitted per year	Isolates are sent from participating labs to IHMA. IHMA performs AST and interpretation. Data are housed with the Micron Group	CLSI, EUCAST	AST data collected at IHMA and shared with Micro Group for long-term storage	Data are publicly available. The ATLAS program aims to publish these data annually	Challenging logistics between all participants in the surveillance program, and lack of patient data to correlate with isolate testing
GLASS (global in scope)	WHO	GLASS provides criteria to empower each country’s National Coordinating Center (NCC) to manage and monitor their own programs	Voluntary, through partnering countries	GLASS receives data from Member States’ Ministries of Health through yearly data calls	Interpretations only	EUCAST, CLSI, others, depending on the contributing country	Data uploads are automatically validated and checked for inconsistencies. The NCC manages data validation at the national level	WHO Glass shares its data via its online dashboards, databases, and annual reports and publications	Data from the United States are siloed. Globally, it can be difficult and expensive for countries outside of the United States to upgrade their existing AMR surveillance systems. Current AST interpretation-only reporting is complicated by variability in breakpoint use
SENTRY	JMI	Most sites are long-standing partners, but JMI reaches out annually to new/existing partners	Voluntary, through partnership	Annually	Partnering labs send isolates to JMI, and the AST is performed by JMI	CLSI, EUCAST, US FDA	Data are internally validated by trained staff to see if data make sense or if further testing is warranted	Data are publicly available on JMI’s website	None identified by interviewee or literature review
NHSN	CDC	Healthcare facilities can enroll online at https://www.cdc.gov/nhsn/enrollment/index.html	Participation is voluntary	Monthly	MICs and interpretations. However, facilities have the option to report interpretations only	CLSI	NHSN provides guidance documents for data validation on an annual basis	Data are shared publicly via annual reports	Manual data entry is not available for the AR option, and strict vendor software requirements may limit facilities’ ability to participate, which leads to unreported AMR data
CDC ARLN and EIP	CDC	Clinical labs are not currently part of ARLN. Instead, they report through public health labs in each state, which are selected based on geographic representation and laboratory capacity	Participation is voluntary	If a jurisdiction has the capacity, they submit data automatically. Otherwise, data are submitted monthly	MICs only	CLSI, or FDA if there is not a CLSI breakpoint available	The data get standardized at the state public health department level	Data are publicly shared via annual reports	HL7, a standard for exchanging information between medical information systems, is not accessible to all states. This is an interoperability challenge
Gonococcal Isolate Surveillance Project (GISP)	CDC	Participation is achieved through existing ARLN relationships	Participation is voluntary	Specimens are collected from the first 25 men testing positive for urethral gonorrhea each month	Specimens only	CLSI	AST results are collated and analyzed by CDC GISP	Data are shared publicly. GISP releases reports regularly that are available for download	By leveraging participation through existing ARLN relationships, GISP is prone to the same data transfer accessibility limitations
NTSS	CDC	Lab participation is required – this is a reportable pathogen	Required	Case by case basis	Isolates from clinical labs to public health labs	N/A	N/A	Data are shared publicly via annual reports including trends, risk factors, treatment, and resistance. The public can also run and export reports via OTIS	Measurement of drug resistance for TB may come from molecular and phenotypic methods. Culture is slow for mycobacteria and requires special laboratory infrastructure and PPE (BSL-3)
NARMS	CDC	Clinical labs send select enteric isolates to their public health lab as they are isolated	Participation is voluntary for clinical labs	In real time, as isolates are collected and identified	Public health labs submit select enteric isolates from clinical specimens from humans to CDC NARMS for AST.	Various, dependent on each lab’s preference. Furthermore, results and interpretation can be changed any time, even years after data are reported	CDC NARMS has a series of lab validation approvals for AST and WGS. Their approval is mandatory for isolate resistance results to be released	Data are shared publicly and available in various formats via the NARMS Now website: NARMS Now (cdc.gov)	Variability in the use of interpretive criteria by individual labs may make data generalizability or interpretation challenging

Note. AMR, antimicrobial resistance; AST, antimicrobial susceptibility testing; ATLAS, Antimicrobial Testing Leadership and Surveillance; CDC, Centers for Disease Control and Prevention; CDC ARLN, CDC Antimicrobial Resistance Laboratory Network; CDC EIP, CDC Emerging Infections Program; CLSI, Clinical and Laboratory Standards Institute; EUCAST, European Committee for Antimicrobial Susceptibility Testing; GISP, Gonococcal Isolate Surveillance Project; GLASS, Global Antimicrobial Resistance and Use Surveillance System; HL7, Health Level 7; IHMA, International Health Management Associates; MIC, Minimum Inhibitory Concentration; NARMS, National Antimicrobial Resistance Monitoring System; NCC, National Coordinating Center; NHSN, National Healthcare Safety Network; NTSS, National Tuberculosis Surveillance System; OTIS, Online Tuberculosis Information System; PPE BSL-3, Personal Protective Equipment Biosafety Level 3; TB, tuberculosis; US FDA, US Food and Drug Administration; WGS, whole genome sequencing; WHO, World Health Organization.

### JMI labs SENTRY program

Jones Microbiology Institute, Inc (JMI) is an independent laboratory providing AMR surveillance and drug development services, with Sentry as its prevalence-based antibiotic and antifungal surveillance program.^29–36^ JMI collaborates annually with laboratories worldwide, recruiting sites each January. Participating sites send the first 40 isolates for various infection types (eg, bloodstream, skin and soft tissue, respiratory urinary, and inter-abdominal), and JMI conducts AST, publishing MICs and interpretations on their dashboard following CLSI, EUCAST, or US FDA guidelines. Data publication usually occurs early in the year after isolate receipt and AST (Table 1).

### CDC programs

The CDC runs multiple surveillance programs, each contributing to AMR surveillance efforts in unique ways. These programs include National Healthcare Safety Network (NHSN), NARMS for Enteric Bacteria, ARLN, Emerging Infections Program (EIP), GISP, and the National Typhoid and Paratyphoid Fever Surveillance (NTPFS) program. NHSN is the most commonly used healthcare-associated infection tracking system in the United States and serves over 25,000 healthcare facilities.^37^ The NHSN Antimicrobial Use and Resistance (AUR) module supports clinical laboratory AST data sharing, although various data exchange requirements must be met to participate.^18^ The NARMS program is one of the longest-standing CDC surveillance programs and tracks changes in AST data for enteric gram-negative organisms isolated from humans, retail meats, and food animals in the United States. Data from the NARMS program have been used to support studies assessing changes in resistance patterns in enteric bacteria^38,39^ as well as comparisons between AMR detection methods for enteric pathogens.^40,41^ NTPFS collects data on typhoid fever since 1975 and paratyphoid since 2008. Clinically identified cases are reported to local health departments, and laboratory isolates with case details are sent to the CDC. NARMS at the CDC houses enteric AMR data, aiding investigations, clinical guidance, and public health interventions.^42–44^

The EIP, a CDC collaboration with state health departments, consists of 10 core programs. One such program, Healthcare-Associated Infections – Community Interface (HAIC), focuses on infections and AMR in healthcare settings, gathering data from NHSN and collaborating with healthcare facilities and state public health programs.^45^ GISP, a collaboration between sexually transmitted infection clinics and ARLN labs, collects monthly specimens from the first 25 men with urethral gonorrhea. After completing AST and interpreting breakpoints according to CLSI guidelines, partnering ARLN laboratories send results to the CDC for analysis, informing various epidemiology studies over the years.^46–51^

## Global AMR surveillance programs with US participation

### Antimicrobial testing leadership and surveillance (ATLAS)

Antimicrobial testing leadership and surveillance (ATLAS), Pfizer’s AMR surveillance program, offers an open-access database containing raw, anonymized patient MICs and metadata. Established in 2004 as the Tigecycline Evaluation Surveillance Trial (TEST),^52^ ATLAS collaborates with laboratories, often through long-standing relationships, and identifies new partners based on recommendations from its field teams. When establishing new sites, ATLAS coordinates with International Health Management Associates (IHMA), an independent laboratory. Partnering laboratories voluntarily send approximately 280 isolates per year to IHMA for AST, MIC measurement, and gold standard interpretations. The program aims to publish data every 6–8 months, potentially extending to 12 months or more based on context. The publicly available ATLAS data can be accessed by clinicians, researchers, or the general public to understand AMR trends regionally and globally (Table 1).

### World Health Organization GLASS program

The Global Antimicrobial Resistance and Use Surveillance System (GLASS) was initiated by WHO to standardize global AMR data, addressing previous challenges in varied data collection methods. GLASS collaborates with countries with existing national surveillance platforms, guiding them in setup, even in early implementation stages without AMR data. Participating countries submit data annually through GLASS, following AST interpretations from CLSI, EUCAST, or US FDA guidelines. GLASS validates, analyzes, and publishes data annually. Learnings are shared through regular reports, publications, and an online dashboard, allowing segmentation by region, infectious syndrome, and antimicrobial group. GLASS is planning individual rollouts to enhance data quality by specifying sources like intensive care unit or outpatient settings (Table 1).

### Current barriers to successful AMR surveillance in the United States

AMR surveillance is evolving across local, regional, and national levels. However, the foundation described above faces several barriers to achieving greater success. The accessibility of high-quality data is paramount to the accuracy and reliability of burden estimates,^53,54^ but selection bias is inherent in our surveillance systems, from the patient level (eg, where isolates are tested) to the system level (eg, where results are reported).^53^ Lacking far-reaching and inclusive data crossing the patient care continuum, AMR burden estimates depend on modeling.^1,55^

Starting in 2024, the NHSN AUR module data will be required to be submitted for eligible hospitals that participate in the Centers for Medicare and Medicaid Services Promoting Interoperability Programs.^56^ This may prove challenging for healthcare providers unfamiliar with this system, and leveraging existing relations with other members of the team who submit data to NHSN, such as infection prevention and control, could be of value. Once access to the NHSN data is established, Antimicrobial Stewardship Programs will have to recognize that the data themselves will not drive change, rather how they are deployed at the institution will.

Silos of microbiology laboratory data and clinical patient outcomes limit the conclusions that can be drawn about the impact of changes in AMR.^57^ Data privacy rules, while critical, influence the linkage of these 2 data streams. As of 2021, 96% of non-federal acute care hospitals and 4 in 5 office-based physicians have adopted a certified EHR, but only 59% of long-term care hospitals possess this technology.^58^ Finally, although this study focuses on human health, surveillance across the One Health Approach^59^ is an area that is currently not well explored.

### Future directions for AMR surveillance in the United States

As the United States continues to confront the challenge of AMR, several key areas emerge as focal points for advancing AMR surveillance.

#### Modernizing healthcare institutions with EHRs

The integration of EHRs in healthcare institutions is not just a technological upgrade but rather a transformative step toward more efficient and effective AMR surveillance. Healthcare institutions have increasingly adopted EHRs and are using them for more advanced purposes in recent years.^60^ As shown in this narrative, EHRs have the potential to enable the rapid collection, analysis, and sharing of critical data related to antimicrobial use and resistance patterns. This real-time data stream is invaluable for early detection of emerging resistance trends, facilitating prompt and targeted responses. However, the interoperability of EHRs across different healthcare institutions is a major barrier to widespread applications in AMR surveillance. A robust, generalizable EHR AMR surveillance application could enhance the comprehensiveness of surveillance efforts, allowing for a more coordinated response to AMR. Additional challenges such as ensuring data privacy and standardizing data formats must be addressed to fully harness the potential of EHRs in AMR surveillance.

#### Leveraging implementation science

Implementation science in AMR surveillance involves a strategic approach to integrating new practices within healthcare systems. This growing field focuses on evaluating the feasibility, acceptability, appropriateness, and cost-effectiveness of interventions, which are vital for their long-term success.^61^ By using an implementation science framework, AMR surveillance strategies can be designed to be adaptable and responsive to the dynamic nature of AMR. Continuous assessment and modification of strategies, based on ongoing data analysis and feedback, are essential to ensure effectiveness across different healthcare environments. Moreover, selecting the right methods, guided by tools like the Consolidated Framework for Implementation Research and the Expert Recommendations for Implementing Change process, is crucial for effective integration.^62^ This approach ensures that AMR surveillance strategies are not only sustainable but also continuously evolve with changing healthcare needs and evidence.

#### Whole genome sequencing for AST prediction

WGS represents a significant advancement in the field of AMR surveillance. WGS has the potential to rapidly and accurately identify resistance mechanisms, offering a level of precision and predictive power that traditional methods cannot match.^63–65^ The implementation of WGS in clinical settings could revolutionize how healthcare providers approach antimicrobial therapy, leading to more tailored and effective treatment strategies. However, the integration of WGS into standard practice faces regulatory challenges, including the need for comprehensive validation, standardization of data interpretation, and ensuring compatibility with existing laboratory workflows.^65^ Addressing these barriers is essential to fully realize the benefits of WGS in enhancing AMR surveillance capabilities.

## Conclusions

AMR surveillance in the United States demands multi-level collaboration and a unified approach with standardized procedures, requiring modernized healthcare and integrated EHRs for streamlined data sharing. Implementation science should be leveraged to ensure that AMR surveillance programs are feasible and sustainable across various environments. Future studies should focus on identifying clinical laboratory processes for AMR surveillance participation, as well as the application and utility of advanced technologies such as data analytics and WGS.
